# Artificial Intelligence and Psychiatric Training: Opportunities, Challenges, and the Future of Mental Health Education

**DOI:** 10.7759/cureus.115140

**Published:** 2026-08-25

**Authors:** Nazar Muhammad, Genna Sharp, Ankit Gautam, Sagarika Ray

**Affiliations:** 1 Psychiatry, Nassau University Medical Center, East Meadow, USA; 2 Psychiatry and Behavioral Sciences, Hofstra University, Uniondale, USA; 3 Psychiatry and Behavioral Sciences, Nassau University Medical Center, East Meadow, USA

**Keywords:** artificial intelligence, chatgpt, graduate medical education, large language models, medical education, mental health, psychiatry education, residency training

## Abstract

Artificial intelligence (AI) is rapidly transforming healthcare delivery, research, and medical education. Within psychiatry, advances in machine learning, natural language processing, large language models (LLMs), and AI-enabled simulation are reshaping clinical practice and trainee education. Educational and clinical applications include personalized learning, virtual patient encounters, clinical decision support, longitudinal electronic health record (EHR) synthesis, automated feedback, and documentation assistance. These tools may strengthen diagnostic reasoning, psychopharmacology education, psychotherapy training, and administrative efficiency while allowing more time for patient-facing care. However, algorithmic bias, misinformation, privacy, academic integrity, cost, and overreliance may undermine independent clinical judgment. These risks are especially important for novice learners and in child and adolescent assessment, where chronological age does not capture cognitive development, cultural context, or nonverbal and affective cues. Empirical outcome data evaluating formal AI curriculum implementation in psychiatry residency and fellowship programs remain limited, with few studies assessing effects on trainee competence, clinical reasoning, error recognition, patient safety, or sustained clinical performance. This narrative review examines current applications, limitations, and ethical considerations and proposes a staged, competency-based curriculum in which trainees establish core interviewing, observation, and diagnostic skills before progressing to supervised clinical AI use. AI should augment, rather than replace, human interpretation, therapeutic relationships, and professional accountability. Accordingly, this review is primarily directed toward graduate medical education leaders, psychiatry residency and fellowship program directors, and clinical educators who are responsible for the development, implementation, and oversight of competency-based AI curricula within psychiatric training programs.

## Introduction and background

Artificial intelligence (AI) has emerged as one of the most influential technologies in modern healthcare. Advances in machine learning, natural language processing, deep learning, and generative AI have expanded digital systems beyond traditional data analysis toward complex tasks involving clinical reasoning, content generation, documentation, and education. Large language models (LLMs), AI models trained on extensive text datasets to interpret and generate natural language, have accelerated interest in how AI may reshape health professions education, including curriculum design, self-directed learning, simulation, assessment, and clinical decision support [1-6].

Psychiatry occupies a unique position in this technological evolution. Unlike specialties that rely heavily on laboratory testing, imaging, or procedural skills, psychiatric practice is grounded in communication, behavioral observation, empathy, therapeutic alliance, cultural formulation, and contextual interpretation of patient experience. This makes psychiatry both highly suitable and uniquely vulnerable to AI-assisted education. AI may help trainees practice psychiatric interviewing, generate clinical vignettes, review psychopharmacology, and receive immediate feedback. At the same time, the limitations of AI are especially important in psychiatry because subtle emotional cues, narrative meaning, risk assessment, and relational trust remain central to clinical care [1-3,7].

Recent psychiatry-specific reviews suggest that AI is beginning to affect psychiatric education through case-based learning, simulation, educational resource development, clinical skills training, assessment, and academic integrity challenges [1-3]. However, the evidence base remains formative. Many applications are early-stage, proof-of-concept, or extrapolated from broader medical education literature. More specifically, empirical outcome data evaluating the implementation of formal AI curricula in psychiatric residency and fellowship programs remain limited, including whether such curricula improve trainee competency, preserve independent clinical reasoning, enhance patient safety, or improve clinical outcomes. Consequently, psychiatric educators need practical frameworks that address not only what AI can do, but also how trainees should learn to evaluate and supervise AI outputs in clinical practice.

This narrative review examines current and emerging applications of AI in psychiatric training, including AI-assisted learning, virtual patient simulation, clinical decision support, longitudinal electronic health record synthesis and measurement support, psychopharmacology education, psychotherapy training, and child and adolescent psychiatry, including digital phenotyping, which refers to the use of data from smartphones, wearable devices, and other digital interactions to characterize behavioral patterns relevant to mental health. The review also examines the use of AI in healthcare administration and proposes a competency-based framework for preparing future psychiatrists to practice safely and effectively in an AI-augmented mental health system.

Methods

A narrative review methodology was used to synthesize literature relevant to AI and psychiatric education. Searches were conducted in databases including PubMed, Google Scholar, and Scopus. Search terms included “artificial intelligence,” “generative artificial intelligence,” “large language models,” “ChatGPT,” “psychiatry education,” “psychiatric training,” “residency education,” “graduate medical education,” “simulation-based learning,” “psychotherapy training,” “mental health education,” “digital phenotyping,” “electronic health records,” “longitudinal data,” “measurement-based care,” “suicide risk prediction,” and “AI ethics.”

Priority was given to English-language articles published from 2023 through 2026, with earlier foundational sources included when relevant. Included sources consisted of scoping reviews, systematic reviews, narrative reviews, original studies, expert commentaries, professional guidance, and medical education frameworks. Articles were selected if they addressed AI applications in psychiatry, psychology, mental health care, medical education, graduate medical education, simulation, clinical decision support, ethics, digital mental health, or child and adolescent mental health. Because the field remains heterogeneous and rapidly evolving, findings were organized thematically rather than analyzed by meta-analysis.

## Review

Current applications of AI in psychiatric training

AI is increasingly being integrated into psychiatric education through applications that support learning, assessment, clinical reasoning, simulation, and administrative efficiency. Current applications can be grouped into AI-assisted learning, virtual patient simulations, clinical decision support, longitudinal EHR synthesis and measurement support, psychopharmacology education, psychotherapy training, child and adolescent psychiatry training, and healthcare systems education.

A scoping review of AI in psychiatric education from 2016 to 2024 identified major themes including the need for AI curricula, AI-generated educational resources, clinical skills development, assessment, academic integrity, and tensions between innovation and traditional educational priorities [1]. Another scoping review of generative AI in psychiatric education found potential roles in case-based learning, simulation, content synthesis, and assessment, while emphasizing concerns about accuracy, bias, security, and privacy [2].

AI-assisted learning

Traditional psychiatric education relies on lectures, textbooks, journal clubs, clinical supervision, and direct patient care. These methods remain essential, but AI-assisted learning may help individualize education. LLMs can generate board-style questions, summarize complex topics, explain psychopharmacology concepts, create differential diagnosis exercises, and produce practice cases for residents and fellows. Broader medical education reviews have described potential uses of LLMs in personalized learning, curriculum development, question generation, and clinical reasoning practice [4-6].

AI may also help trainees manage information overload. Psychiatry residents are expected to follow rapidly expanding literature on psychopharmacology, neuroscience, psychotherapy, neurodevelopmental disorders, suicide prevention, and healthcare systems. AI tools can assist with literature screening, article summarization, guideline comparison, and generation of learning objectives. However, trainees must learn that AI-generated summaries may omit key limitations or produce fabricated information. Therefore, AI-assisted learning should be paired with verification against peer-reviewed literature, clinical guidelines, and faculty supervision [5,6,8].

A single-center randomized trial of 129 first-year medical students found no significant difference in overall clinical-reasoning scores between AI chatbot-generated and expert-written feedback immediately after the intervention or 10 days later; however, expert feedback produced higher scores for complicated cases [9]. Because the study involved preclinical learners and one nonpsychiatric topic, its findings support cautious use of AI feedback as an adjunct for routine formative learning rather than equivalence to expert supervision in psychiatric training. Whether AI-generated longitudinal psychiatric histories can accelerate the development of trainee expertise remains untested.

Virtual patient simulations

Simulation-based education is particularly relevant to psychiatry because residents must develop interviewing skills, risk assessment skills, crisis management skills, and therapeutic communication. AI-powered virtual patients can simulate depressive episodes, mania, psychosis, anxiety, substance use disorders, trauma-related symptoms, neurodevelopmental conditions, personality pathology, and emergency presentations. These encounters can be repeated and modified to vary symptom severity, cultural background, developmental stage, and clinical complexity [1-3].

Virtual patients may help trainees practice suicide risk assessment, motivational interviewing, capacity evaluation, de-escalation, and diagnostic formulation before encountering high-risk clinical situations. AI simulations may reduce dependence on costly standardized patient programs and provide immediate formative feedback. However, many current tools operate primarily through text or speech and cannot reliably integrate the full mental status examination. Silence, gaze, psychomotor activity, facial expression, incongruent affect, and changes in interpersonal engagement may alter a clinician's formulation even when the spoken content is unchanged. Multimodal systems may detect some observable cues, but detection is not equivalent to contextual understanding. Virtual patients should therefore supplement, not replace, standardized patients, supervised clinical encounters, and faculty feedback [2,7,10].

Clinical decision support systems

Clinical decision-support systems use computational models to assist diagnosis, prognosis, risk stratification, treatment selection, and care coordination. In psychiatry, potential applications include suicide risk prediction, relapse prediction, medication response modeling, hospitalization risk assessment, and symptom monitoring. For trainees, decision-support systems may serve as educational tools by allowing comparison between resident-generated formulations and data-driven recommendations.

However, psychiatric decision-making involves uncertainty, longitudinal context, collateral information, culture, trauma history, family systems, medical comorbidity, and patient preference. AI tools may miss these contextual factors or overemphasize patterns present in training data. Reviews of machine learning for suicide prediction show promise but also highlight limitations in validation, bias, calibration, and clinical implementation [11,12]. Psychiatry trainees should therefore learn how to interpret model outputs probabilistically and ethically rather than treating them as definitive clinical answers.

Longitudinal EHR synthesis and measurement-based care

Clinically important information is often dispersed across physician notes, nursing documentation, laboratory results, imaging, and medication records, making longitudinal chart review inefficient and vulnerable to omission. Deep-learning models have demonstrated the technical feasibility of generating hospital-course summaries from multi-document inpatient EHR data [13]. In a study of 100 general internal medicine hospitalizations, an EHR-integrated LLM required less resident editing than physician-generated summaries (31.5% vs 44.8%) and was rated more complete and similarly concise and cohesive by attending physicians; however, the LLM summaries contained significantly more confabulations [14]. These findings support clinician-LLM collaboration for documentation, not autonomous summarization, and make verification essential.

Longitudinal analysis is particularly relevant to psychiatry because symptoms, treatment response, and risk may unfold over months or years. Models using routinely collected EHR data have predicted nonfatal suicide attempts in adolescents across time windows from one week to two years [15]. Another study trained on records from 41,721 children and adolescents reported areas under the receiver operating characteristic curve (AUCs) of 0.81 to 0.86 across prediction horizons from the index encounter to one year [16]. These results demonstrate predictive feasibility, but they do not establish that deployment improves outcomes, prevents suicide attempts, or can substitute for direct assessment, collateral information, and clinical judgment.

Longitudinal measurement is also central to measurement-based care (MBC), in which standardized symptom measures are collected repeatedly and used to guide treatment. In adults with major depressive disorder, a randomized controlled trial found faster symptom improvement and higher response and remission rates with MBC than with standard care [17]. A meta-analysis of seven randomized trials involving 2,019 participants associated MBC with lower endpoint symptom severity, higher remission, and better medication adherence, while the pooled response-rate difference was not statistically significant; certainty was limited by the small number of trials, substantial heterogeneity, and risk of bias [18]. MBC therefore has an evidence base in adult depression, but it is not itself an AI intervention. Whether AI-derived insights from broader EHR data add incremental benefit remains unproven.

AI in psychopharmacology education

Psychopharmacology is one of the most demanding areas of psychiatric training. Residents must master medication mechanisms, pharmacokinetics, drug-drug interactions, adverse effects, monitoring requirements, treatment algorithms, and shared decision-making. AI systems can support interactive medication learning by generating comparison tables, explaining mechanisms of action, creating treatment algorithms, and simulating medication-management scenarios.

AI-assisted tools may support case-based learning, content synthesis, interactive practice, and formative feedback in psychiatric education [1,2,5-7]. Within psychopharmacology training, these general educational functions may be applied to supervised medication-comparison, drug-interaction, and monitoring exercises. These tools may also help trainees recognize drug interactions and organize monitoring schedules. Nevertheless, AI-generated prescribing suggestions must be treated cautiously. Psychopharmacology decisions require individualized assessment of diagnosis, medical comorbidity, side-effect vulnerability, patient preference, safety risk, and access barriers.

AI in psychotherapy training

Psychotherapy training emphasizes empathy, emotional attunement, reflective listening, alliance formation, transference and countertransference awareness, and flexible response to patient narratives. AI systems may assist psychotherapy education through role-play simulations, motivational interviewing practice, cognitive behavioral therapy exercises, dialectical behavior therapy skills rehearsal, supportive psychotherapy scenarios, and feedback on communication style.

The limitations are equally important. AI-based avatars can support dialogue practice, but text- or audio-dominant systems may not register silence, gaze, psychomotor change, facial expression, or incongruent affect. Even multimodal systems detect patterns rather than understand the patient's subjective experience. The therapeutic alliance is not reducible to correct words or structured techniques, and AI cannot fully reproduce human presence, emotional resonance, ethical accountability, or the lived relational experience of therapy. Psychiatric training programs should therefore frame AI as a practice and feedback tool, not as a replacement for supervision or psychotherapy with real patients [10,19,20].

AI in child and adolescent psychiatry training

Child and adolescent psychiatry provides an especially important area for AI education. AI methods are being studied for autism spectrum disorder screening, ADHD assessment, youth suicide prediction, social media analysis, digital phenotyping, and early identification of mood and anxiety symptoms. Digital phenotyping uses data from smartphones, wearables, and digital interactions to infer behavioral patterns, sleep, activity, social engagement, and possible mental health risk [21,22].

These technologies may improve early detection and monitoring, but pediatric applications raise heightened ethical and developmental concerns. Chronological age is not a reliable substitute for cognitive, language, or social-emotional development; an output labeled appropriate for a 15-year-old may be unsuitable for an adolescent functioning at a different developmental level. AI may also misinterpret communication or behavior when culture, family norms, disability, trauma, and language are incompletely represented. Children and adolescents may not fully understand data collection, long-term privacy risks, algorithmic profiling, or how digital data could affect access to care. Training should therefore require individualized developmental and cultural formulation, parent consent, adolescent assent, and direct clinical observation rather than accepting age-matched output at face value [10,19-23].

AI in utilization review and healthcare administration

Psychiatrists increasingly participate in healthcare administration, quality improvement, utilization review, population health, and prior authorization processes. AI may streamline administrative workflows through chart summarization, documentation support, coding assistance, medical necessity screening, and predictive analytics. When accurate and appropriately supervised, these tools may reduce clerical burden and allow clinicians to devote a greater proportion of the encounter to patient communication, observation, and education.

At the same time, the use of AI in coverage decisions or resource allocation requires strong safeguards. If algorithms influence access to inpatient care, residential treatment, medications, or psychotherapy, transparency and appeal mechanisms are essential. Training programs should expose residents to healthcare systems, value-based care, quality metrics, documentation standards, and ethical oversight of AI in administrative settings. The goal should be augmentation of clinician review, not replacement of professional judgment.

The principal applications of AI in psychiatric training are summarized in Table 1. The potential educational benefits and risks of AI integration are compared in Table 2.

**Table 1:** Current applications of AI in psychiatric training Reference sources for the table: General guidance and competency frameworks [24-26], medical and health professions education [27-29], mental health applications [30], and governance guidance [31]. Reference sources by domain: AI-assisted learning [1-9]; virtual patients [1-3,7,10]; clinical decision support [11,12]; longitudinal synthesis and measurement [13-18]; psychopharmacology [1,2,5-7]; psychotherapy training [10,19,20]; child and adolescent psychiatry [15,16,21-23]; administration [13,14,24-26,31]. ADHD, attention-deficit/hyperactivity disorder; AI, artificial intelligence; CBT, cognitive behavioral therapy; DBT, dialectical behavior therapy; EHR, electronic health record; MBC, measurement-based care

Domain	Examples	Educational benefit
AI-assisted learning	Board-style questions, article summaries, study plans	Personalized and efficient learning
Virtual patients	Psychiatric interviews, suicide risk assessment, de-escalation scenarios	Deliberate practice in low-risk settings
Clinical decision support	Risk prediction, differential diagnosis, treatment pathways	Clinical reasoning and systems awareness
Longitudinal synthesis and measurement	EHR summarization, trajectory review, MBC support	Practice integrating longitudinal data while verifying AI outputs
Psychopharmacology	Medication comparisons, monitoring reminders, interaction checks	Safer and more structured prescribing education
Psychotherapy training	Role-play, CBT/DBT skills practice, motivational interviewing	Communication practice and formative feedback
Child and adolescent psychiatry	Autism screening, ADHD assessment, digital phenotyping	Developmentally and culturally informed assessment practice
Administration	AI-assisted documentation, utilization review, quality metrics	Less clerical burden and more patient-facing time, if validated

**Table 2:** Potential benefits and risks of artificial intelligence integration in psychiatric education Reference sources by topic: personalized learning and hallucinations [4-8]; access, bias, and inequity [8,19,23,24,30,31]; simulation and overreliance [1-3,7,10,25,26]; feedback, privacy, and confidentiality [2,7-10,24,31]; documentation and academic integrity [1,2,13,14,24]; systems-based practice and humanistic concerns [10,19,20,24-26]; patient-facing time and documentation accuracy [13,14]; scalability, cost, and unequal access [1-8,24-26,31]; longitudinal synthesis and confabulation risk [13,14].

Potential benefits	Potential risks
Personalized learning	Hallucinations and misinformation
Expanded access to educational tools	Algorithmic bias and inequity
Simulation of rare or high-risk cases	Overreliance and cognitive offloading
Immediate feedback	Privacy and confidentiality concerns
Documentation support	Academic integrity concerns
Support for systems-based practice	Reduced humanistic focus if poorly implemented
Potentially more patient-facing time	Inaccurate notes or hidden correction burden
Scalable simulation and educational access	Licensing, infrastructure costs, and unequal access
Faster synthesis of longitudinal records	Confabulations or omitted context requiring clinician verification

AI competencies for the future psychiatrist

As AI becomes embedded in clinical workflows, psychiatric training programs should develop competencies that extend beyond traditional medical knowledge. Several medical education organizations have emphasized responsible AI use, including the need for governance, transparency, fairness, and educator preparedness [24-26]. For psychiatry, these competencies must also protect the relational and humanistic core of the specialty. Broader medical education literature also emphasizes implementation planning, faculty development, and institutional readiness [27-29].

First, trainees need AI literacy. They should understand basic concepts such as machine learning, natural language processing, LLMs, predictive analytics, algorithmic bias, hallucination, model validation, and data privacy [24-27,29]. Second, trainees need prompt engineering and effective human-AI interaction skills. The quality and safety of AI outputs often depend on how questions are framed, what context is provided, and whether limitations are requested [5-7,24-26]. Third, trainees need critical appraisal skills. They must evaluate AI outputs for accuracy, relevance, bias, evidence quality, and applicability to the patient in front of them [5-8,24-26].

Fourth, trainees require digital professionalism. They must know when not to enter patient information into nonapproved systems, how to disclose AI use when appropriate, and how to preserve patient confidentiality [24-26,31]. Fifth, trainees should develop human-AI collaboration skills. AI may help generate differential diagnoses or educational materials, but responsibility for clinical judgment remains with the psychiatrist [24-26]. Finally, trainees need ethical oversight skills, including awareness of bias, informed consent, equity, and accountability [19,23-26,30,31].

Proposed core AI competencies for psychiatry trainees are summarized in Table 3.

**Table 3:** Proposed AI competencies for psychiatry trainees Reference sources by competency: AI literacy [24-27,29]; prompt engineering [5-7,24-26]; critical appraisal [5-8,24-26]; digital professionalism [24-26,31]; human-AI collaboration [24-26]; ethical oversight [19,23-26,30,31]; contextual formulation [10,19,21-23]. AI, artificial intelligence; LLM, large language model

Competency	Description	Training example
AI literacy	Understanding basic AI concepts and limitations	Introductory seminar on LLMs and machine learning
Prompt engineering	Communicating effectively with AI systems	Generate and critique differential diagnoses
Critical appraisal	Evaluating accuracy, bias, and evidence quality	Compare AI output with guidelines
Digital professionalism	Using AI while protecting confidentiality	Policy-based documentation exercises
Human-AI collaboration	Using AI as an adjunct to clinical judgment	Supervised AI-assisted case formulation
Ethical oversight	Recognizing bias, fairness, consent, and accountability	Case conference on AI and patient safety
Contextual formulation	Integrating development, culture, language, and nonverbal behavior	Critique an age-matched AI assessment using a full formulation

Opportunities for psychiatric education

AI presents several educational opportunities. It can expose learners to diverse and rare clinical presentations, provide immediate feedback, support deliberate practice, and expand access to educational materials. AI-generated cases can be adjusted to include different developmental stages, cultural contexts, comorbidities, and levels of acuity. This may improve diagnostic reasoning and reduce dependence on opportunistic clinical exposure.

Personalized learning is another major opportunity. Adaptive systems can identify gaps in psychopharmacology, psychotherapy, emergency psychiatry, child psychiatry, or systems-based practice and generate individualized learning plans. AI may also support residents in resource-limited settings by providing access to structured educational support that may otherwise be unavailable. In global psychiatry, such tools could help expand mental health training capacity, provided they are implemented with cultural adaptation and quality control.

Administrative efficiency is a potential educational benefit, but it should be measured rather than assumed. Programs evaluating AI-assisted documentation should assess total cost of ownership, time saved, note accuracy, correction burden, clinician well-being, patient acceptance, and whether saved time is actually redirected to patient care or supervision. Cost-effectiveness and equitable access should be treated as implementation outcomes, not presumed advantages [24-26].

Discussion

AI represents a major technological development in psychiatric education. The current evidence base is early but growing, with recent scoping reviews documenting AI applications in psychiatric teaching, learning, simulation, assessment, and educational resource development [1-3]. Broader medical education literature supports the potential of AI and LLMs to assist with personalized learning, case generation, question writing, and curriculum support, while also warning about hallucinations, bias, academic integrity, and the need for responsible governance [4-8,24-26].

Recent evidence sharpens the distinction between technically feasible components and demonstrated clinical or educational benefit. AI can synthesize complex records [13,14], longitudinal EHR models can estimate youth suicide risk [15,16], MBC improves selected outcomes in adult depression [17,18], and generative AI can support routine clinical-reasoning feedback [9]. However, these separate findings do not establish that an AI-enabled longitudinal workflow improves psychiatric outcomes, overcomes interoperability barriers, or accelerates psychiatric expertise. They are best understood as plausible components that require prospective implementation studies.

The central argument of this review is that psychiatry should not approach AI as a replacement technology, but as an educational and clinical augmentation tool requiring supervision, critical appraisal, and ethical boundaries. This distinction is especially important because psychiatric diagnosis depends on both elicited content and direct observation. Empathy, alliance, trust, meaning-making, cultural humility, and interpretation of nonverbal affective cues cannot be outsourced to an algorithm.

A useful organizing principle is competence before clinical dependence. Early residents should learn to obtain a history, perform a mental status examination, integrate developmental, cultural, and contextual data, and construct a diagnosis and plan without automated substitution. AI can then be introduced through progressive entrustment: first as an object of critique, later as a supervised aid, and finally as a monitored clinical tool when the trainee can identify errors and remain accountable [25,26].

**Figure 1:**
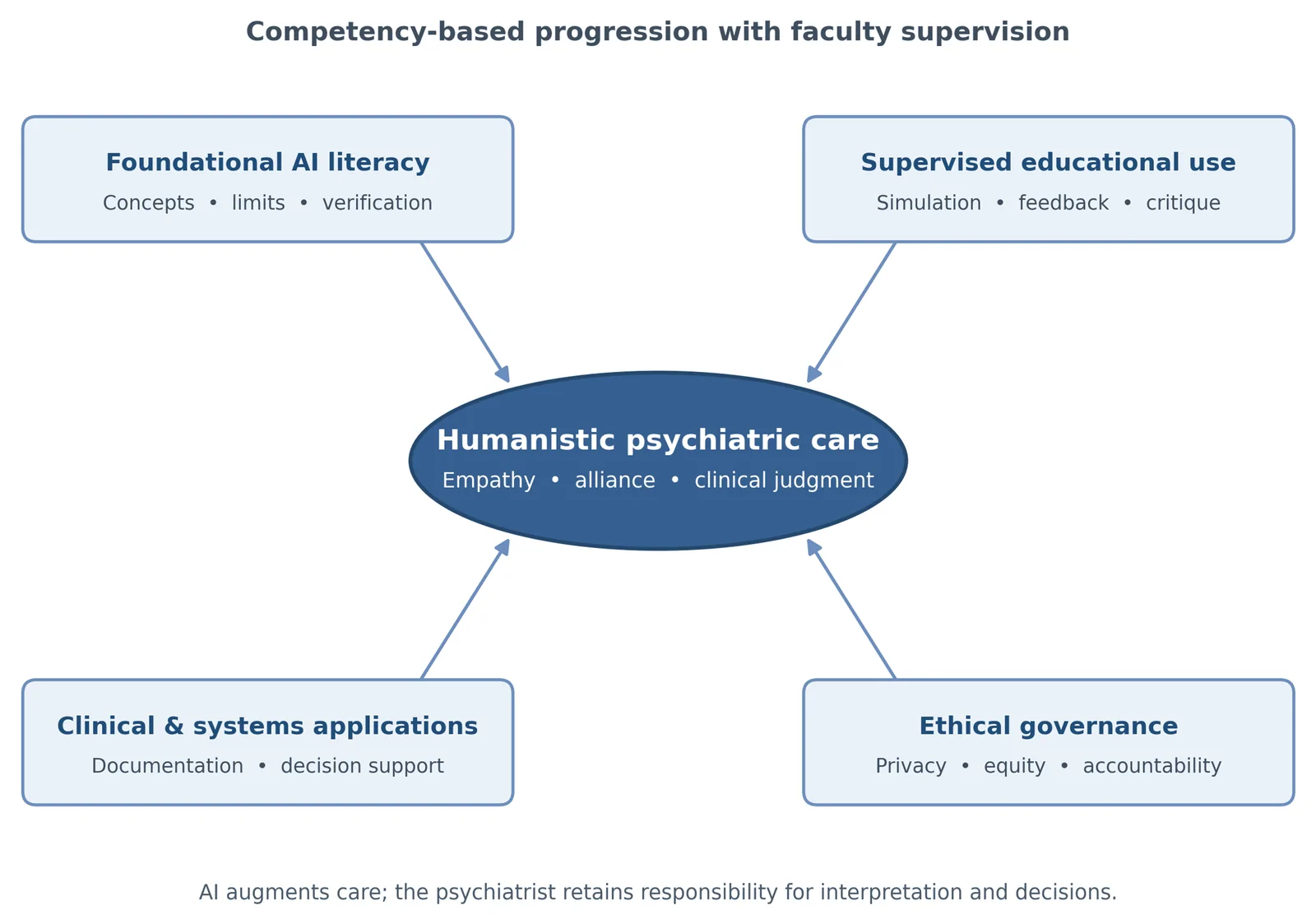
Human-centered, competency-based approach Four coordinated domains foundational AI literacy, supervised educational use, clinical and systems-based applications, and ethical governance surround humanistic psychiatric care. Progression from education to clinical use should be competency-based and supervised, while responsibility for interpretation and clinical decisions remains with the psychiatrist. This author-created conceptual model was informed by professional guidance and competency-based medical education frameworks [24-26,31]. AI, artificial intelligence.

This human-centered, competency-based approach is illustrated in Figure 1. This approach does not reject efficiency. AI-assisted documentation may reduce clerical work and increase time available for patient interaction, teaching, and reflection. However, educational value, time savings, correction burden, total cost, and equity should be evaluated prospectively. The goal is not maximal AI use; it is appropriate use that improves learning and care while preserving the psychiatrist's responsibility for interpretation and decisions.

Challenges and Limitations

Reliability is a central limitation of AI in psychiatric education. LLMs may generate plausible but inaccurate statements, fabricated references, or incorrect clinical recommendations that novice learners may not recognize [5-8,10,23].

Overreliance is particularly concerning for novice learners who have not yet developed an independent standard against which to evaluate AI output. Programs should therefore require residents to complete their own assessments before reviewing AI suggestions, introduce AI-assisted clinical use only after competence has been demonstrated, and establish clear boundaries for AI use in assignments and assessments [5,7,25,26].

This narrative review also has methodological limitations. It was not conducted as a systematic review or meta-analysis, and because the literature was not identified through a systematic search and selection process, some relevant studies may not have been captured. The field is rapidly changing, and new tools, studies, and regulatory standards continue to emerge. Many current AI applications in psychiatric education remain experimental, and high-quality longitudinal studies evaluating trainee competency, patient outcomes, and safety are limited. In addition, some recommendations in this manuscript are based on synthesis and expert interpretation rather than mature outcomes data.

Ethical Considerations

Ethical AI integration in psychiatric education requires attention to transparency, accountability, equity, privacy, consent, and professional integrity. Transparency means that trainees and supervisors should understand when AI is being used and recognize its limitations. Accountability means that final responsibility for patient care remains with the clinician. Equity requires ongoing evaluation of whether AI systems perform fairly across different demographic, linguistic, cultural, and socioeconomic groups. Because psychiatric records contain particularly sensitive information, training programs should restrict patient data to approved platforms and establish clear rules governing data use, storage, access, and confidentiality [19,23,30,31].

Informed consent may become increasingly important as AI tools are integrated into clinical care, documentation, and patient communication. Patients may reasonably expect to know whether AI contributed to clinical notes, educational materials, or recommendations. Professional integrity also requires clear standards for authorship, plagiarism, and disclosure when AI is used in scholarly writing or educational work.

Recommendations for Residency and Fellowship Programs

Psychiatric training programs should replace unstructured AI use with a staged, competency-based approach. The following principles operationalize that approach: (i) Foundational AI literacy should be established. Model limitations, hallucination, bias, privacy, security, and source verification should be taught before clinical application; (ii) Independent clinical competence is required. Early training should prioritize direct interviewing, mental status examination, diagnostic reasoning, risk assessment, and developmental and cultural formulation. Trainees should complete their own assessment before viewing AI output; (iii) Progressive entrustment should be used. Starting with deidentified educational exercises and output critique, one should advance to supervised documentation or decision support only after observed competence; progression should depend on performance rather than training year [25,26]; (iv) Observation and relationship should be preserved. AI simulations and summaries should supplement standardized patients, direct care, and supervision; they should not replace nonverbal observation, therapeutic alliance, or patient preferences; (v) Governance and faculty oversight should be created. Approved platforms, prohibited data, disclosure, review, audits, and escalation pathways should be created, and faculty should be prepared to supervise AI-assisted work [24-26,31]; (vi) Value and equity should be measured. Learning, errors, documentation quality, time, cost, patient acceptance, and patient-facing time should be measured. Equity should be monitored across developmental, cultural, linguistic, and socioeconomic groups.

A proposed staged AI curriculum and suggested use boundaries across residency and fellowship training are presented in Table 4.

**Table 4:** Proposed staged AI curriculum for psychiatry residency and fellowship training This proposed competency-based curriculum is the authors’ synthesis informed by professional guidance and medical education frameworks [24-26,31]. AI, artificial intelligence; PGY, postgraduate year

Training level	Curriculum focus	Suggested use boundary
PGY-1	Foundations, privacy, bias, and independent interviewing	Deidentified learning tasks; complete the interview, mental status examination, and differential before viewing AI output
PGY-2	Output critique, psychopharmacology, and documentation principles	Supervised exercises with source verification and faculty review; no unsupervised diagnostic substitution
PGY-3	Psychotherapy, child psychiatry, and clinical decision support	Supervised clinical integration after demonstrated competence; preserve direct observation and contextual formulation
PGY-4	Leadership, quality improvement, research, Iimplemntation and governance	Monitored clinical integration after demonstrated competency; conduct audits and error reviews, evaluate outcomes, and supervise junior learners while retaining responsibility for clinical decisions.
Fellowship	Specialty-specific implementation and scholarship	Outcomes evaluation and supervision within specialty-specific safeguards

Future Directions

Future research should evaluate whether AI-assisted psychiatric education improves measurable outcomes such as diagnostic accuracy, interviewing skills, suicide risk assessment, psychopharmacology competence, psychotherapy skills, documentation quality, and trainee confidence. Studies should compare AI simulation with standardized patients; distinguish outcomes for novice and advanced trainees; and test whether supervised AI use strengthens or weakens independent clinical reasoning. Evaluations of documentation and longitudinal synthesis tools should include time saved, correction burden, confabulations, total cost, clinician well-being, patient acceptance, and whether efficiencies increase patient-facing time. Research should also determine whether AI can reconcile longitudinal data across noninteroperable health systems, whether AI-supported risk detection leads to earlier effective intervention and better patient outcomes, and whether longitudinal synthesis improves trainee expertise rather than merely reducing chart-review time. Child and adolescent psychiatry deserves special attention because tools must be evaluated across developmental levels, languages, cultures, family contexts, and disability. Multimodal systems should be tested for whether they add valid information from nonverbal cues without encouraging false confidence. Professional organizations should develop psychiatry-specific guidance on AI literacy, ethics, disclosure, progressive entrustment, and clinical supervision.

## Conclusions

AI is poised to transform psychiatric education and clinical practice. AI-driven educational tools offer opportunities to enhance learning, simulation-based training, clinical decision support, psychopharmacology education, psychotherapy practice, child psychiatry training, and administrative efficiency. However, concerns regarding bias, misinformation, privacy, academic integrity, and overreliance require careful attention.

Psychiatric residency and fellowship programs should adopt structured, staged, and competency-based approaches to AI education. Trainees should establish independent interviewing, observation, developmental and cultural formulation, and diagnostic skills before progressing to supervised clinical AI use. The future of psychiatric training will not depend on replacing psychiatrists with AI. It will depend on preparing psychiatrists to work effectively alongside AI while preserving empathy, therapeutic relationships, ethical judgment, clinical reasoning, and professional accountability.
